# Supplementary material for: Intrinsically disordered intracellular domains control key features of the mechanically-gated ion channel PIEZO2
Source: Nat Commun. 2022 Mar 15;13:1365. doi: 10.1038/s41467-022-28974-6 (PMC8924262; doi:10.1038/s41467-022-28974-6)
Supplement: Supplementary file 1 — Supplementary Information [file 41467_2022_28974_MOESM1_ESM.pdf]

Supplementary Information

Title: „Intrinsically disordered intracellular domains control key features of the mechanically-gated ion channel PIEZO2"

Authors

Clement Verkest[1†], Irina Schaefer[2†], Timo A. Nees[2,3], Wang Na[2], Juri M. Jegelka[2], Francisco J. Taberner[2,4], Stefan G. Lechner[1,2,*]

Affiliations

[1] Department of Anesthesiology, University Medical Center Hamburg-Eppendorf, Martinistrasse 52, 20246 Hamburg, Germany

[2] Institute of Pharmacology, Heidelberg University, Im Neuenheimer Feld 366, 69120 Heidelberg, Germany

[3] Clinic for Orthopedics and Trauma Surgery, Center for Orthopedics, Trauma Surgery and Spinal Cord Injury, Heidelberg University Hospital, Schlierbacher Landstrasse 200a, 69118 Heidelberg, Germany

[4] current address: Instituto de Neurosciencias de Alicante, Universidad Miguel Hernández – CSIC, Alicante, Spain

[†] these two authors contributed equally to this work

*correspondence: s.lechner@uke.de

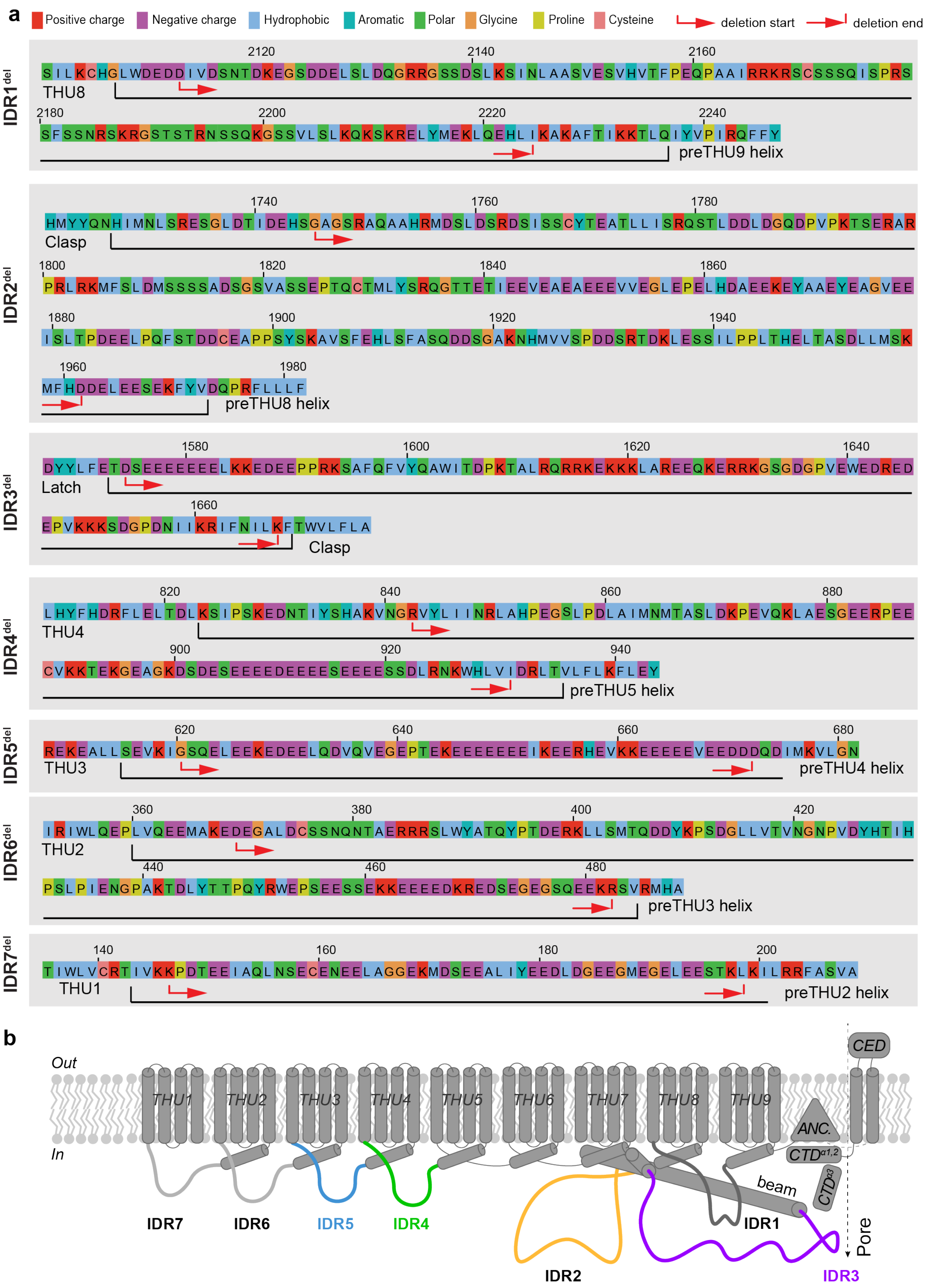

Supplementary Fig. 1, PIEZO2-IDR amino acid sequences (related to Fig. 1) .
a, Color-coded (top) amino acid sequence of PIEZO2-IDR1-7. The positions of the IDRs are indicated by the black brackets below the sequences and start and end position of the regions that were deleted are marked with red arrows. **b**, Cartoon depicting the membrane topology of PIEZO2 and the positions of the seven IDRs.

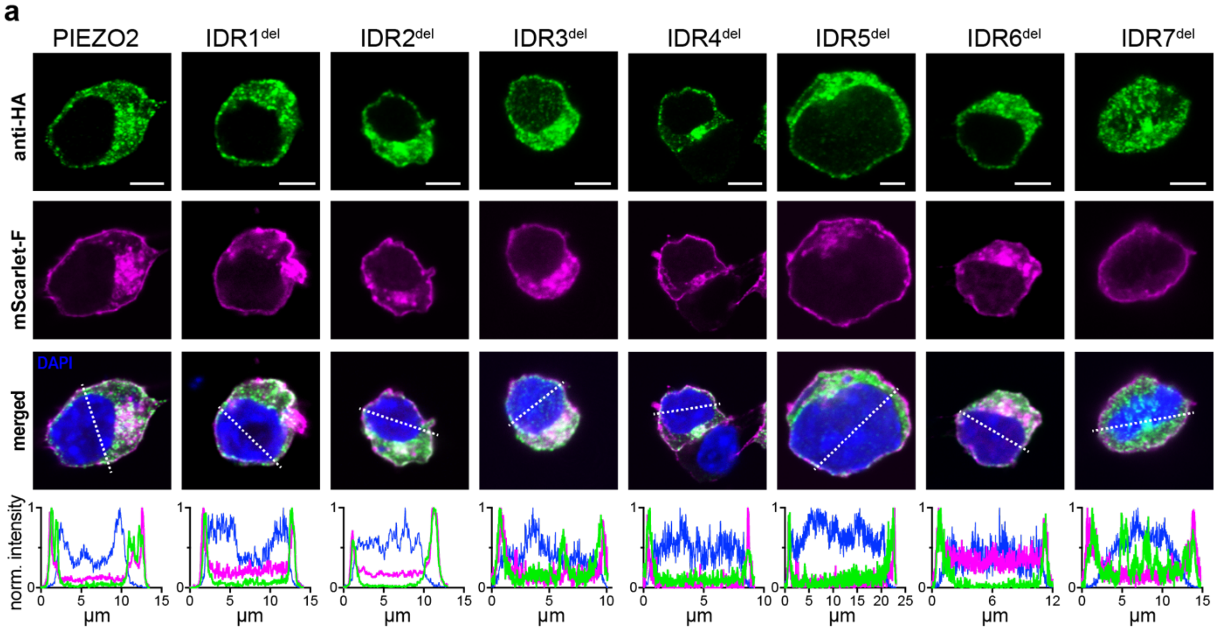

Supplementary Fig. 2, Membrane expression of PIEZO2 and IDR-deletions (related to Fig. 1).

a, Representative immunofluorescent images showing the subcellular localization of PIEZO2 and IDR1-7^{del}. N2A-P1KO expressing PIEZO2 and IDR1-7 were stained for PIEZO2 (HA tag, green, top) and the nucleus (DAPI, blue, bottom). The plasma membrane was visualized with endogenous mScarlet-F fluorescence (magenta, middle, see method section for details). Scale bar in top row equals 5 µm. PIEZO2 and IDR1-7 expression in the plasma membrane was determined by performing line scans along the dashed lines (merged images). Line scans of the shown images normalized to the maximum intensity in each channel are shown in the bottom row. The following numbers of cells were analyzed: PIEZO2 (N=22), IDR1^{del}(N=22), IDR2^{del}(N=23), IDR3^{del}(N=7), IDR4^{del}(N=27), IDR5^{del}(N=30), IDR6^{del}(N=17), IDR7^{del}(N=10),

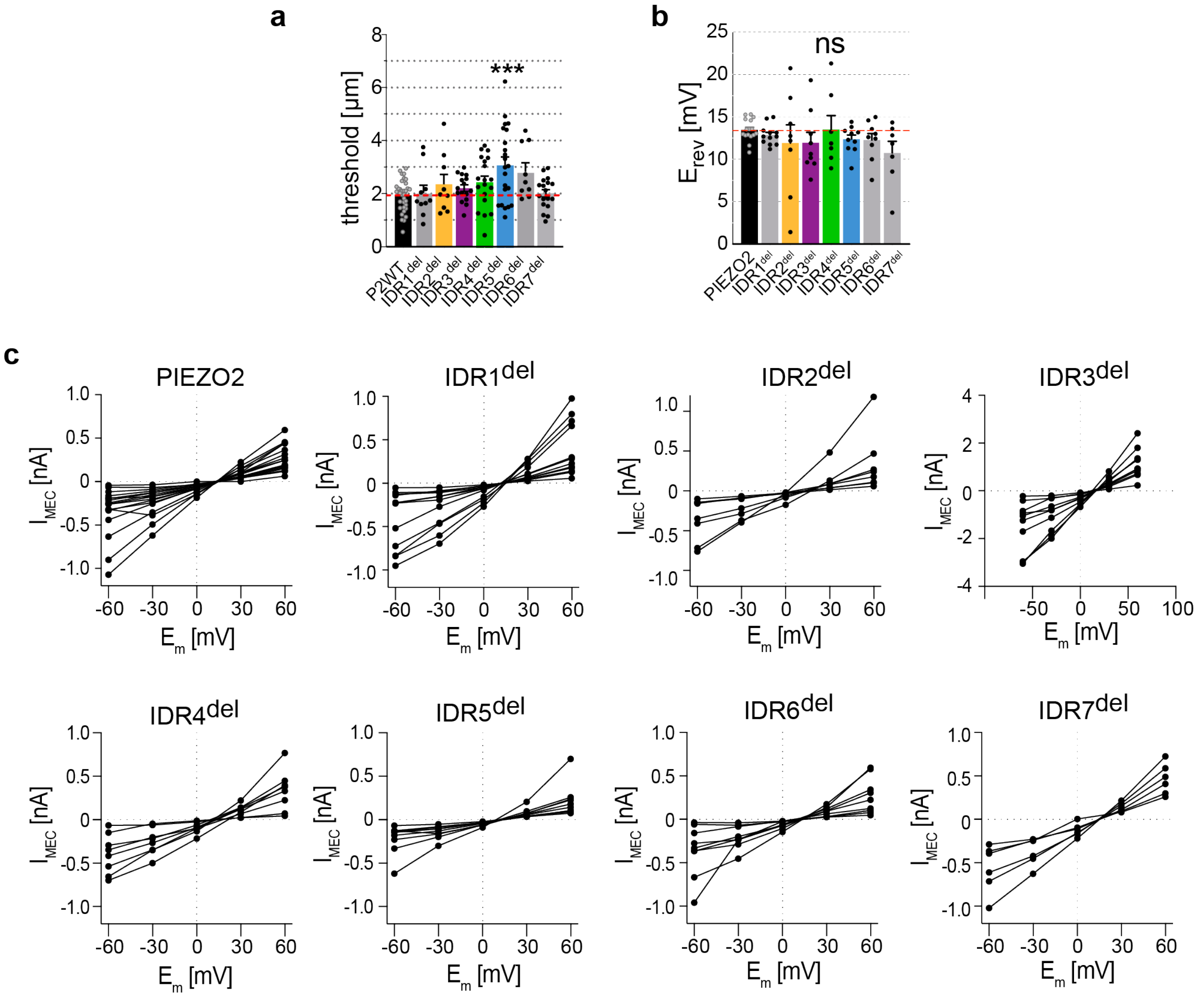

Supplementary Fig. 3, Deletion of IDRs does not alter current-voltage relationships (related to figure 1)

a, Comparsion of the mean ± s.e.m activation threshold of mechanically activated currents mediated by PIEZO2 and IDR^{del} mutant. Comparison with One-Way ANOVA, F(7,130)=4.093, Dunnett's post-test PIEZO2 vs IDR5^{del}, ***p<0.0001. **b**, Reversal potential (E_{rev}) of PIEZO2 and IDR^{del}. Bar graphs are mean ± s.e.m., with individual values. Comparison with Kruskal-Wallis, ns p=0.4886. *n* per group are (from left to right): 16, 12, 8, 9, 7, 10, 9, 7. **c**, Linear plots of the I-V relationships from individual cells in PIEZO2 and IDR^{del} mutants. Symbols are individual peak current amplitude recorded at the indicated voltage. *n* number of cells per group are identical to **b**.

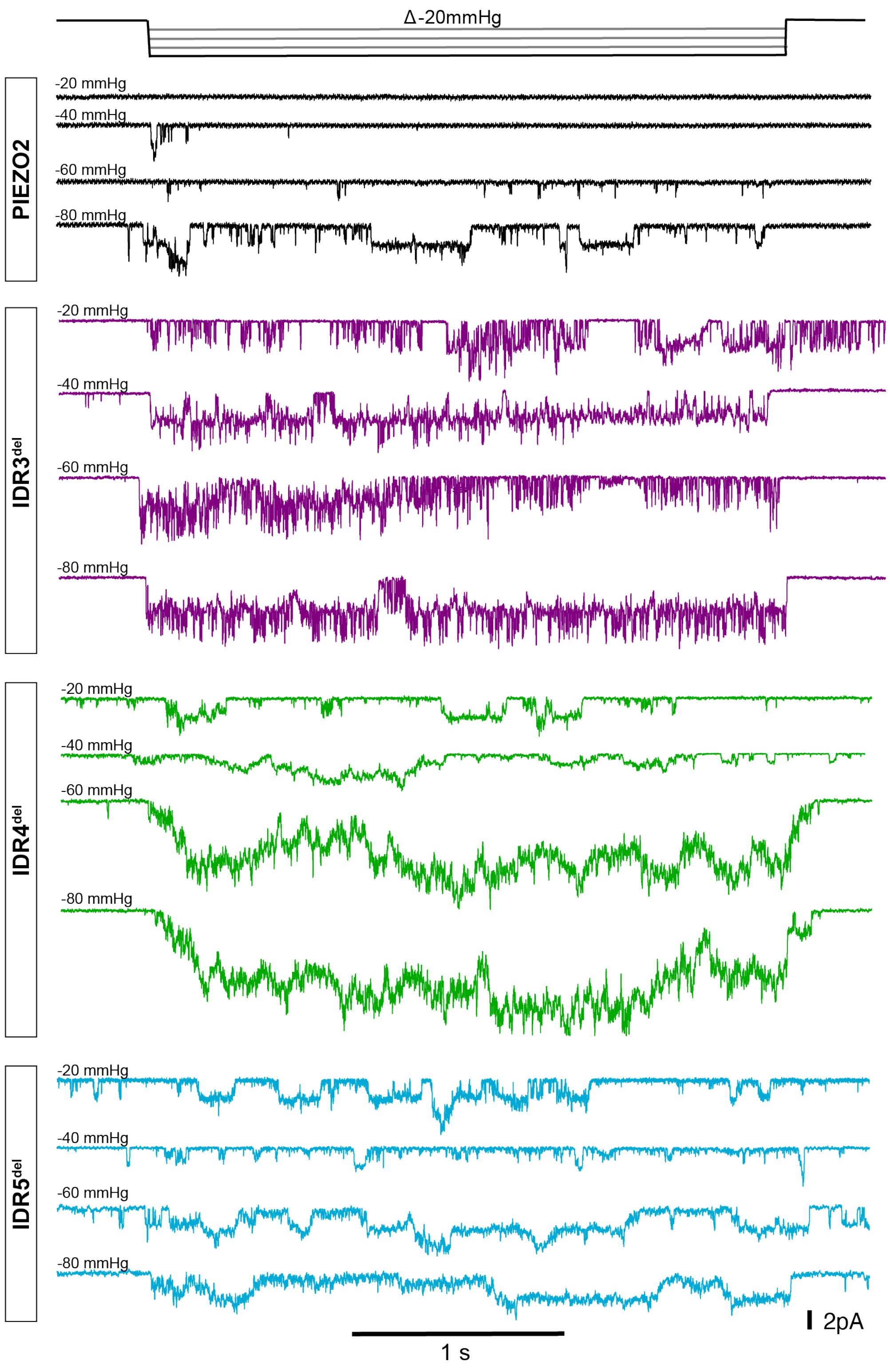

Supplementary Fig. 4, Pressure-response relationship of PIEZO2, IDR3^{del}, IDR4^{del} and IDR5^{del} (related to figure 2).
Representative example traces of PIEZO2, IDR3^{del}, IDR4^{del} and IDR5^{del}-currents evoked by membrane stretch evoked by increasing pressure stimuli (-20 mmHg, -40 mmHg, -60 mmHg and -80 mmHg).

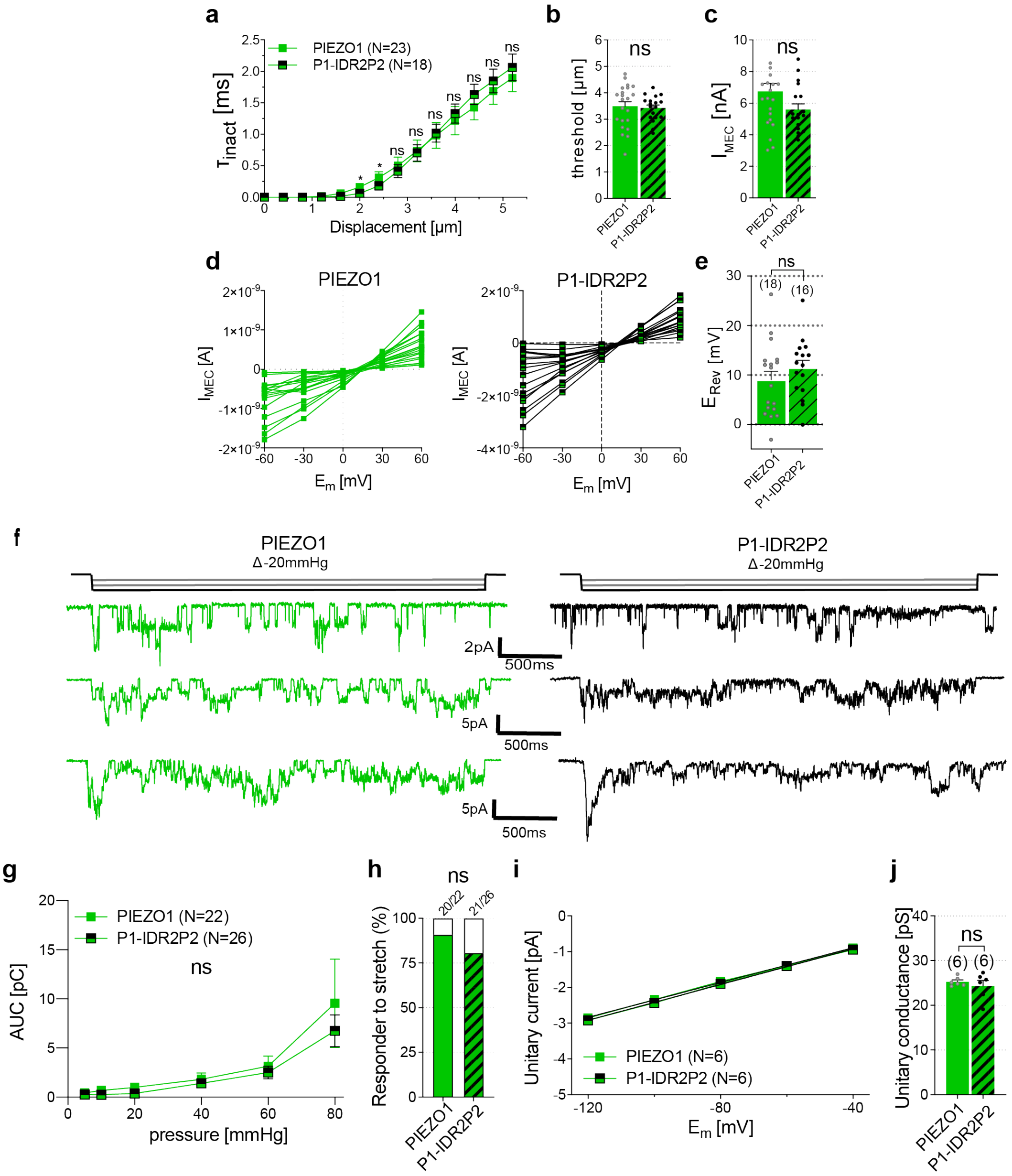

Supplementary Fig. 5, Electrophysiological characterization of PIEZO1 and PIEZO1-IDR2P2 (related to figure 3). a, Displacement-responses curves of peak amplitudes of poking-evoked PIEZO1 (N=23) and P1-IDR2P2 (N=18) currents in N2a-P1KO cells. Symbols represent means ± s.e.m. Comparison with Mann Whitney test (ns = not significant; * $P_{2\mu m}$=0.0381; * $P_{2.4\mu m}$=0.0356) **b**, Comparison of the mechanical activation thresholds of PIEZO1 (N=23) and PIEZO1-IDR2P2 (N=18) mutant in the poking assay using Unpaired t-test (ns; p=0.6274). Bars represent means ± s.e.m. and values from individual cells are shown as filled circles. **c**, Comparison of the inactivation time constant (τ_{inact}) of PIEZO1 (N=23) and P1-IDR2P2 (N=18) currents with Unpaired t-test (ns, p=0.0901). **d**, I-V relationships of PIEZO1 (left, green) and P1-IDR2P2 (right, black) currents plotted separately for recordings from individual cells. **e**, Comparison of the reversal potential (E_{rev}) of PIEZO1 and P1-IDR2P2 determined by linear regression of the plots shown in **d**. Bar graphs represent mean ± s.e.m. and E_{rev}-values from individual cells are shown as filled circles. N-numbers are indicated in brackets above the bars. Comparison with two-sided unpaired t-test (ns, p=0.2973). **f**, Representative example traces of stretch-activated single-channel currents at -100mV evoked by stimuli of increasing negative pressure from N2a-P1KO cells expressing of PIEZO1 (left, green) and P1-IDR2P2 (right, black). **g**, Pressure-responses curves of PIEZO1 and P1-IDR2P2. Symbols are mean ± s.e.m. Comparison with Mann Whitney test. N-numbers of cells per group are indicated in the graph legend. **h**, Comparison of the proportion of cells responding to stretch amongst cells expressing PIEZO1 (20 from 22 tested cells) and P1-IDR2P2 (21 from 26 tested cells). Fisher's exact test (ns, p=0.429). **i**, I-V relationships of pressure-evoked PIEZO1 and PIEZO1-IDR2P2 currents. Symbols are mean ± s.e.m. N-numbers are the same as in **j. j**, Unitary conductance of PIEZO1 and P1IDR2P2. Bar graphs are mean ± s.e.m., with individual values. Comparison with Mann Whitney test (ns, p>0.999).

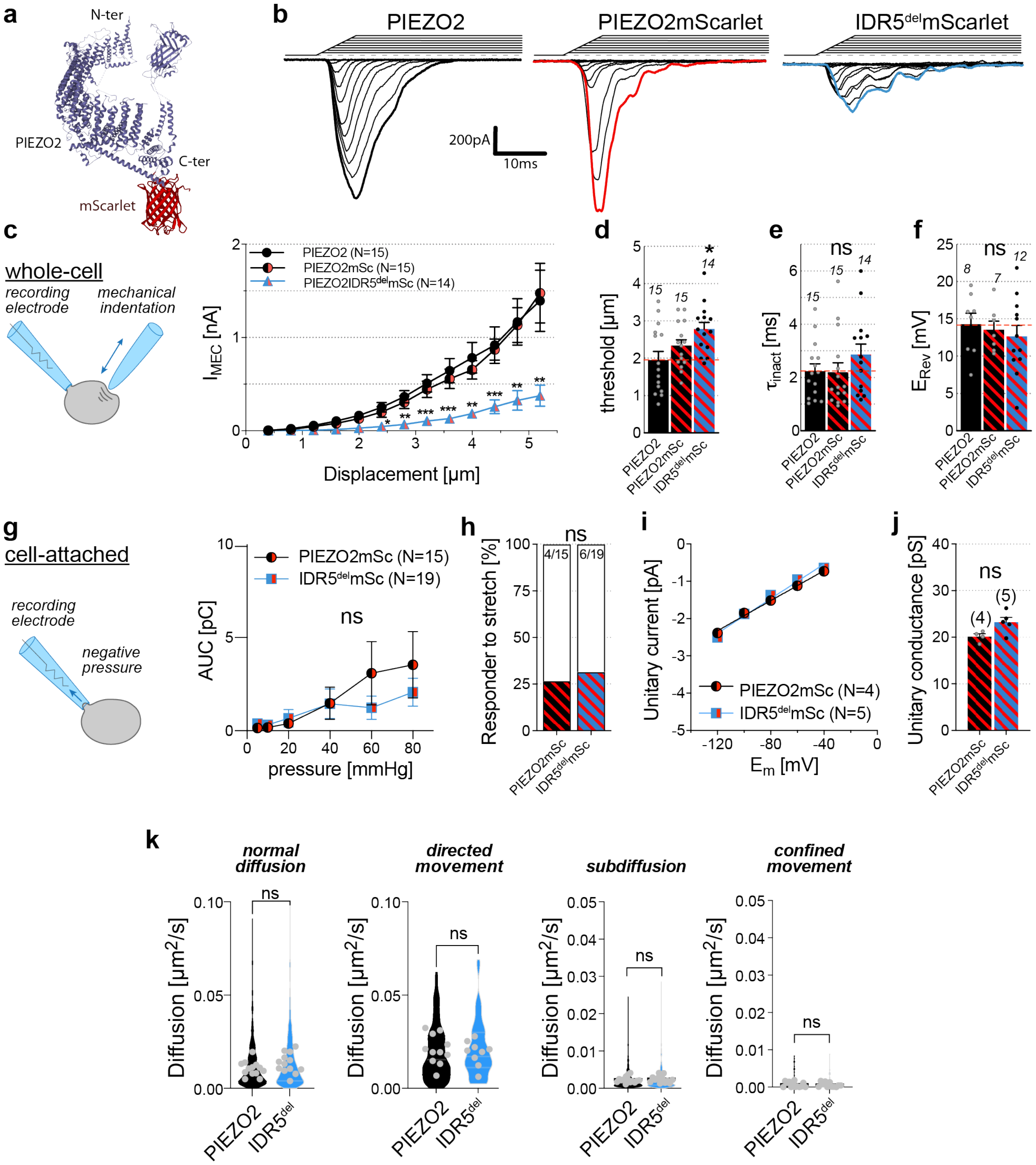

Supplementary Fig. 6, Electrophysiological characterization of mScarlet-tagged PIEZO constructs (related to figure 4). a, Cartoon depicting the structure of a PIEZO2 protamer (6KG7) C-terminally tagged with the red fluorescent protein mScarlet. **b**, Representative example traces of mechanically-evoked whole-cell currents mediated by PIEZO2 (left), PIEZO2mScarlet (middle) and IDR5^{del}mScarlet (right), recorded at -60mV. **c**, Recording configuration cartoon (left) and displacement-responses curves (right) of the ± s.e.m. peak current amplitudes of PIEZO2 (N=15) , PIEZO2mScarlet (N=15) and IDR5^{del}mScarlet (N=14) in N2a-P1KO cells. Mean amplitudes were compared with Kruskal-Wallis test followed by Dunn's post-test. P-values of Dunn's post-test PIEZO2mSc vs. PIEZO2IDR5^{del}mSc (from left to right): *0.01, **0.0018, ***0.0008, ***0.0006, **0.0011, ***0.0006, **0.0012, **0.0013. **d**, Mechanical activation threshold of PIEZO2, PIEZO2mScarlet and IDR5^{del}mScarlet. Comparison with One-way ANOVA, F(2, 41)=4.539. Dunnett's post-test PIEZO2 vs IDR5^{del}mScarlet, * p=0.0121 **e**, Comparison of the mean ± s.e.m. inactivation time constants (τ_{inact}) of PIEZO2 (N=15), PIEZO2mScarlet (N=15) and IDR5^{del}mScarlet (N=14) using Kruskal-Wallis test (ns; p=0.2963). **f**, Comparison of the mean ± s.e.m. Reversal potential (E_{rev}) of PIEZO2 (N=8), PIEZO2mScarlet (N=7) and IDR5^{del}mScarlet (N=12) using one-way ANOVA (F(2,24)=0.3423, ns; p=0.7136). **g**, Recording configuration cartoon (left) and pressure-responses curves (right, total charge

transferred (AUC) vs. pressure) of PIEZO2mScarlet (circles, N=15) and IDR5^{del}mScarlet (squares, N=19). Symbols represent means ± s.e.m. Comparison with two-sided Mann Whitney test. **h**, Comparison of the proportions of cells responding to pressure-induced membrane stretch amongst PIEZO2mScarlet and IDR5^{del}mScarlet-expressing N2a cells using Fisher's exact test (ns p>0.999). **i**, I-V relationships of PIEZO2mScarlet and IDR5^{del}mScarlet. Symbols are mean ± s.e.m. and N-numbers are indicated in the graph legend. **j**, Comparison of the mean ± s.e.m. unitary conductance of PIEZO2mScarlet (N=4) and IDR5^{del}mScarlet (N=5) using Mann-Whitney test (ns p=0.1111). **k**, Violin plots of the diffusion coefficients of individual PIEZO2mScarlet and IDR5^{del}mScarlet clusters from the different track categories: normal diffusion (N_{PIEZO2}=817, $N_{IDR5del}$=622), directed movement (N_{PIEZO2}=117, $N_{IDR5del}$=48), subdiffusion (N_{PIEZO2}=1041, $N_{IDR5del}$=907) and confined movement (N_{PIEZO2}=82, $N_{IDR5del}$=88). The mean diffusion coefficients from each examined cell (PIEZO2, N=13 cells, IDR5^{del}, N=13 cells) are shown as grey circles and compared using Mann-Whitney test.

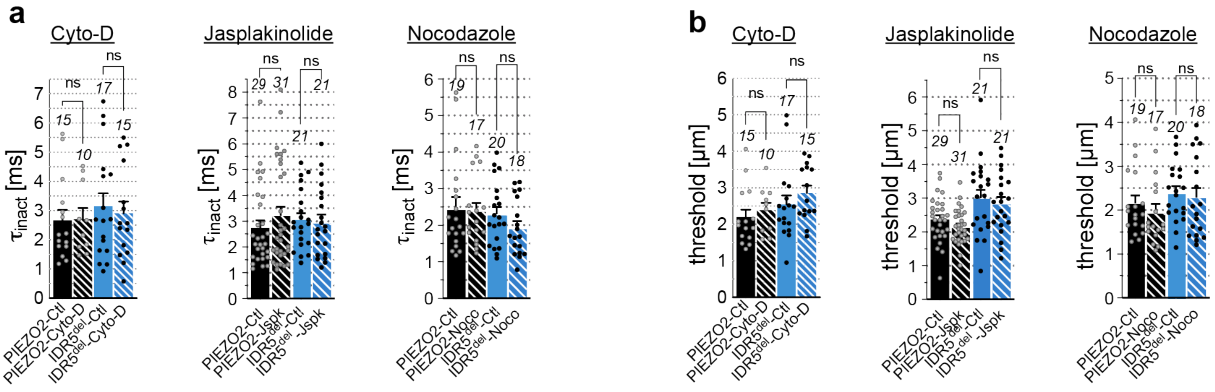

Supplementary Fig. 7 related to figure 5

a-b, Comparison of the inactivation time constants (τ_{inact}, **a**) and mechanical activation thresholds (**b**) of mechanically-evoked PIEZO2 currents (black) and IDR5^{del} currents (blue) recorded in the absence (filled bars) and presence (striped) of cytochalasin-D (Cyto-D, left), Jasplakinolide (middle) and Nocodazole (right). Bars represent means ± s.e.m, N-numbers are indicated above the bars and individual data points are shown as filled circles. Means were compared using the Kruskal-Wallis test.

Supplementary Table 1, Primers used for creating PIEZO2 mutants

Primers	Sequence (5' -> 3')
IDR1_A_FW	GTTACTGTCAACAATGTCagcgctATCCTCGTCCCATAGAC
IDR1_A_RV	GTCTATGGGACGAGGATagcgctGACATTGTTGACAGTAAC
IDR1_B V3 FW	GGTAAACGCCTTTGCTTTagcgctGATTAAATGCTCTTGAAGC
IDR1_B V3 RV	GCTTCAAGAGCATTTAATCagcgctAAAGCAAAGGCGTTTACC
IDR2_A_FW	CCTGGAGCCAGCCCCCGAagcgctGTGCTCGTCGATTGTGTC
IDR2_A_RV	GACACAATCGACGAGCACagcgctTCGGGGGCTGGCTCCAGG
IDR2_B_FW	CAGACTCCTCAAGCTCATCagcgctATCATGGAACATCTTGCTC
IDR2_B_RV	GAGCAAGATGTTCCATGATagcgctGATGAGCTTGAGGAGTCTG
IDR3_A V2 FW	GGAGATTATTATTTGTTTGAAACGAGCGCTGATAGTGAAGAGGAGGAAGAAGAG
IDR3_A V2 RV	CTCTTCTTCCTCCTCTTCACTATCAGCGCTCGTTTCAAACAAATAATAATCTCC
IDR3_B FW	ATATTTAATATCCTGAAGAGCGCTTTCACATGGGTTCTGTTC
IDR3_B RV	GAACAGAACCCATGTGAAAGCGCTCTTCAGGATATTAAATAT
IDR4_A V4 FW	CTATTTATTATTAGGTACACGCGagcgctACCATTGACTTTGGCATGGC
IDR4_A V4 RV	GCCATGCCAAAGTCAATGGTagcgctCGCGTGTACCTAATAATAAATAG
IDR4_B V4 FW	GAGCACAGTGAGCCGGTCagcgctAATCACCAGGTGCCACTTG
IDR4_B V4 RV	CAAGTGGCACCTGGTGATTagcgctGACCGGCTCACTGTGCTC
IDR5_A V2 FW	CTTCAAGTTCTTGACTTCCagcgctAATTTTGACTTCAGAAAG
IDR5_A V2 RV	CTTTCTGAAGTCAAAATTagcgctGGAAGTCAAGAACTTGAAG
IDR5_B V2 FW	GCACCTTCATGATGTCCTGagcgctGTCATCATCTTCCTCCAC
IDR5_B V2 RV	GTGGAGGAAGATGATGACagcgctCAGGACATCATGAAGGTGC
IDR6_A_FW	CAATCCAGGGCACCCTCATCagcgctTTCCTTCGCCATCTCCTC
IDR6_A_RV	GAGGAGATGGCGAAGGAAagcgctGATGAGGGTGCCCTGGATTG
IDR6_B_FW	GGCGTGCATTCTCACACTagcgctCCTTTTCTCCTCCTGGCTC
IDR6_B_RV	GAGCCAGGAGGAGAAAAGGagcgctAGTGTGAGAATGCACGCC
IDR7_A V2 FW	CTGGTCTGCAGAACCATTGTTAAGAGCGCTAAACCAGACACAGAAGAAATAGCC
IDR7_A V2 RV	GGCTATTTCTTCTGTGTCTGGTTTAGCGCTCTTAACAATGGTTCTGCAGACCAG
IDR7_B FW	GAAGAAAGCACAAAACTAAGCGCTAAAATACTTCGCAGGTTC
IDR7_B RV	GAACCTGCGAAGTATTTTAGCGCTTAGTTTTGTGCTTTCTTC
PIEZO1-IDR2P2-A-FW	ggtcccgtggagacccggACCGGTgatggacccagcacagcc
PIEZO1-IDR2P2-A-RV	ggctgtgctgggtccatcACCGGTccgggtctccacgggacc
PIEZO1-IDR2P2-B-FW	gagctgctactggataggcgcACCGGTctgcatatccctgagctggag
PIEZO1-IDR2P2-B-RV	ctccagctcagggatatgcagACCGGTgcgcctatccagtagcagctc
PIEZO2-IDR2-Frag-FW	GCTGAATACCGGTCTTTCCCGAGAATCCGGCCTGGACACAATC
PIEZO2-IDR2-Frag-RV	GCTGAATACCGGTATCATGGAACATCTTGCTCATCAGCAG
PolyA1-FW	GTCAAAATTGGAAGTCAAGAACTTGCAGCAGCGGGAGCTGCGGCGCTCCAAGATGTACAAG TGGAAGG
PolyA1-RV	CCTTCCACTTGTACATCTTGGAGCGCCGCAGCTCCCGCTGCTGCAAGTTCTTGACTTCCAAT TTTGAC
PolyA3-FW	AGGCATGAGGTAAAGAAGGCAGCGGCAGCGGAAGTGGAGGAAGATGAT
PolyA3-RV	ATCATCTTCCTCCACTTCCGCTGCCGCTGCCTTCTTTACCTCATGCCT
PolyA4-FW	AAGGAAGAGGAAGAGGAAGTGGCGGCAGGTGCTGCCCAGGACATCATGAAGGTGCTG
PolyA4-RV	CAGCACCTTCATGATGTCCTGGGCAGCACCTGCCGCCACTTCCTCTTCCTCTTCCTT
IDR3+LatPlugdel-vect-FW	AAAAAAGCAGATGGTCAGGAGTGGAGATTATTATTTGTTTGAAACGAGC
IDR3+LatPlugdel-vect-RV	CAAGTAAGATCTGCAGTGAATGAAATCTGG
IDR3+LatPlugdel-Frag-FW	TTCACTGCAGATCTTACTTGGACATGT
IDR3+LatPlugdel-Frag-RV	TCCTGACCATCTGCTTTTTTTTGCCCTTGGCTTTCTG
