# Supplementary material for: Intrinsically disordered intracellular domains control key features of the mechanically-gated ion channel PIEZO2
Source: Nat Commun. 2022 Mar 15;13:1365. doi: 10.1038/s41467-022-28974-6 (PMC8924262; doi:10.1038/s41467-022-28974-6)
Supplement: Supplementary file 3 — Description of Additional Supplementary Files [file 41467_2022_28974_MOESM3_ESM.pdf]

Description of Additional Supplementary Files

File name: Supplementary Movie 1

Description: TIRF time-lapse imaging showing the lateral diffusion of mScarlet-tagged PIEZO2 clusters. Cluster tracking was performed using TrackMate and individual tracks are shown as yellow lines.

File name: Supplementary Movie 2

Description: TIRF time-lapse imaging showing the lateral diffusion of mScarlet-tagged PIEZO2-IDR5^{del} clusters. Cluster tracking was performed using TrackMate and individual tracks are shown as yellow lines.
