# Supplementary material for: Intrinsically disordered intracellular domains control key features of the mechanically-gated ion channel PIEZO2
Source: Nat Commun. 2022 Mar 15;13:1365. doi: 10.1038/s41467-022-28974-6 (PMC8924262; doi:10.1038/s41467-022-28974-6)
Supplement: Supplementary file 6 — Reporting Summary [file 41467_2022_28974_MOESM6_ESM.pdf]

nature research

Corresponding author(s): Stefan G Lechner

Last updated by author(s): Jan 31, 2022

Reporting Summary

Nature Research wishes to improve the reproducibility of the work that we publish. This form provides structure for consistency and transparency in reporting. For further information on Nature Research policies, see our Editorial Policies and the Editorial Policy Checklist.

Statistics

For all statistical analyses, confirm that the following items are present in the figure legend, table legend, main text, or Methods section.

n/a	Confirmed	
☐	☒	The exact sample size (*n*) for each experimental group/condition, given as a discrete number and unit of measurement
☐	☒	A statement on whether measurements were taken from distinct samples or whether the same sample was measured repeatedly
☐	☒	The statistical test(s) used AND whether they are one- or two-sided<br>*Only common tests should be described solely by name; describe more complex techniques in the Methods section.*
☐	☒	A description of all covariates tested
☐	☒	A description of any assumptions or corrections, such as tests of normality and adjustment for multiple comparisons
☐	☒	A full description of the statistical parameters including central tendency (e.g. means) or other basic estimates (e.g. regression coefficient) AND variation (e.g. standard deviation) or associated estimates of uncertainty (e.g. confidence intervals)
☐	☒	For null hypothesis testing, the test statistic (e.g. *F*, *t*, *r*) with confidence intervals, effect sizes, degrees of freedom and *P* value noted<br>*Give P values as exact values whenever suitable.*
☒	☐	For Bayesian analysis, information on the choice of priors and Markov chain Monte Carlo settings
☒	☐	For hierarchical and complex designs, identification of the appropriate level for tests and full reporting of outcomes
☒	☐	Estimates of effect sizes (e.g. Cohen's *d*, Pearson's *r*), indicating how they were calculated

Our web collection on statistics for biologists contains articles on many of the points above.

Software and code

Policy information about availability of computer code

Data collection	Electrophysiology: Patchmaster (V2x91, HEKA)<br>Immunofluorescence: Leica LAS X (Leica Microsystems)<br>TIRF imaging: NIS Elements (v6.14, Nikon)<br>Western Blots: iBright Imaging Systems (v3.0.1, Invitrogen, ThermoFisher Scientific)
Data analysis	The following sotfwares were used for data analysis and/or collection:<br>FitMaster (V2x91, HEKA)<br>IgorPro 8 (WaveMetrics)<br>Excel (V16.16.27; Microsoft)<br>Prism Graphpad 8 (GraphPad Software)<br>NIS Element (v6.14, Nikon)<br>Fiji/ImageJ (v2.3.0/1.53f; NIH)<br>TrackMate v6.0.1<br>PyMol 2.4.0 (Schrödinger, LLC)<br>iBright Analysis Software (Version 3.0.1; Invitrogen, ThermoFisher Scientific)<br>Jalview 2.11.0<br>PONDR(VSL2) (pondr.com)<br>IUPred (https://iupred2a.elte.hu/)<br>ESPRITZ-N (http://old.protein.bio.unipd.it/espritz/)

For manuscripts utilizing custom algorithms or software that are central to the research but not yet described in published literature, software must be made available to editors and reviewers. We strongly encourage code deposition in a community repository (e.g. GitHub). See the Nature Research guidelines for submitting code & software for further information.

Data

Policy information about availability of data

All manuscripts must include a data availability statement. This statement should provide the following information, where applicable:

- Accession codes, unique identifiers, or web links for publicly available datasets
- A list of figures that have associated raw data
- A description of any restrictions on data availability

All data supporting the findings of this study are available within the article and its supplementary information files. Additional information, relevant data (electrophysiological and imaging raw data) and materials (plasmids encoding the PIEZO channel mutants generated in this study) are available from the corresponding author upon reasonable request. Source data are provided with this paper.

Field-specific reporting

Please select the one below that is the best fit for your research. If you are not sure, read the appropriate sections before making your selection.

☒ Life sciences ☐ Behavioural & social sciences ☐ Ecological, evolutionary & environmental sciences

For a reference copy of the document with all sections, see nature.com/documents/nr-reporting-summary-flat.pdf

Life sciences study design

All studies must disclose on these points even when the disclosure is negative.

Sample size	The two parameters that are crucial for accurate sample size predetermination (variance and effect size) were unknown at the time this study was designed, because it was completely unclear if the deletion of the various IDRs would affect PIEZO2 function and, if so, how big the effects would be. From numerous studies by others and us, in which the same methodology was used, we did, however, know that 10-20 cells per group are sufficient to obtain reliable results from electrophysiological poking experiments and that 3-6 recordings are sufficient to determine single channel conductance, which hardly shows any interexperimental variability.
Data exclusions	For electrophysiological experiments, we excluded from the analyses cells with excessive or unstable leak currents (ex: more than 4pA for single-channel recordings), cells that detached from the coverslips during mechanical stimulation and cells which giga-seals did not withstand at least 7 indentation steps of the probe. For single channel experiments and I/V curves and conductance determination, recordings that displayed non-inactivating responses or unstable openings were not used
Replication	All replication attempts were successful. Electrophysiological experiments were repeated at least on three different cells per day on at least 3 different days, from at least 2 different transfections. Neurite outgrowth experiments were performed on 3 different days, from 3 different transfections. Western-Blot experiments were replicated 4 times, coming from 4 independent transfections. TIRF imaging experiments were performed on cells from 3 different transfections.
Randomization	Randomization is inherent to single cell patch-clamp recordings and for TIRF-imaging from cultured neurons, because all cells in the culture look alike and hence the experimenter inevitably chooses cells without any bias and absolutely random from the pool of healthy, well-attached and fluorescent (transfected) cells. Moreover, for electrophysiological daily experiments, Western-Blot and neurite outgrowth, measurements included the control (PIEZO2/GFP) and mutants (IDRdel). For testing of drugs, daily experiments included the control and treated samples.
Blinding	For logistic reasons the experimenter who performed the N2a cell transfections also had to perform the patch-clamp recordings. Hence, blinding was impossible for electrophysiological data. For the neurite outgrowth experiments and the biotinylation assay the experimenters were, however, blinded during data acquisition and analysis.

Reporting for specific materials, systems and methods

We require information from authors about some types of materials, experimental systems and methods used in many studies. Here, indicate whether each material, system or method listed is relevant to your study. If you are not sure if a list item applies to your research, read the appropriate section before selecting a response.

Materials & experimental systems

n/a	Involved in the study	
☐	☒	Antibodies
☐	☒	Eukaryotic cell lines
☒	☐	Palaeontology and archaeology
☒	☐	Animals and other organisms
☒	☐	Human research participants
☒	☐	Clinical data
☒	☐	Dual use research of concern

Methods

n/a	Involved in the study	
☒	☐	ChIP-seq
☒	☐	Flow cytometry
☒	☐	MRI-based neuroimaging

Antibodies

Antibodies used	-Western-Blot: Rabbit Anti-HA, Sigma-Aldrich Cat# H6908, RRID:AB_260070 / Rabbit Anti-Tubulin (loading control, 1:5000) Sigma-Aldrich Cat# T2200, RRID:AB_262133 / Goat Anti-Rabbit HRP conjugated, Sigma-Aldrich Cat#A6154, RRID:AB_258284<br>-Immunofluorescence: Rabbit Anti-HA, Thermo Fisher Scientific Cat# 71-5500, RRID:AB_2533988 / Donkey Anti-Rabbit AlexaFluor-647, Life technologies, A-31573, RRID:AB_2536183
Validation	For Western-Blot, primary antibodies were succesfully used in the laboratory in a previous study (DOI:10.1073/pnas.1905985116) and are extensively described and validated in multiple other studies (PMID:21830221, PMID:27538435, PMID:27637097, PMID:27725085, PMID:28630333, PMID:28841137, PMID:28951451, PMID:29024645, PMID:29024665, PMID:29073373, PMID:29154130, PMID:29290488, PMID:29290611, PMID:29649442, PMID:29677490, PMID:29681460, PMID:29934346, PMID:29983322, PMID:30044983, PMID:30078703, PMID:30231999, PMID:30244971, PMID:30281024, PMID:30293784, PMID:30404010, PMID:30404013, PMID:30415698, PMID:30550789, PMID:30612859, PMID:30784600, PMID:30886048, PMID:30995488, PMID:31194677, PMID:31235572, PMID:31433974, PMID:31453382, PMID:31549961, PMID:31660580, PMID:31708416, PMID:31904337, PMID:32111743, PMID:32692156, PMID:32778221)<br>For Immunocytochemistry, primary antibody was extensively described and validated in other studies (PMID: 31015432, PMID: 30929979, PMID: 30478286, PMID: 28120887, PMID: 27006476, PMID: 23708797, PMID: 21339294, PMID: 20089652, PMID: 18836075, PMID: 15979566)

Eukaryotic cell lines

Policy information about cell lines

Cell line source(s)	Piezo1 knock-out mouse Neuro2a cells were a gift from Dr. Gary R Lewin (produced from N2a, ATCC CCL-131 from ATCC;Reference: PMID: 29545531)
Authentication	None of the cell lines used were authenticated.
Mycoplasma contamination	Cell line was not tested for mycoplasma contamination
Commonly misidentified lines<br>(See ICLAC register)	No commonly misidentified cell line was used in this study.
